# Vector measurement and performance tuning of a terahertz bottle beam

**DOI:** 10.1038/s41598-018-31250-7

**Published:** 2018-09-04

**Authors:** Heting Li, Xinke Wang, Sen Wang, Wenfeng Sun, Jiasheng Ye, Peng Han, Shengfei Feng, Yue Yu, Yan Zhang

**Affiliations:** 1 Department of Physics, Beijing Key Lab for Metamaterials and Devices, Beijing Advanced Innovation Center for Imaging Technology, Key Laboratory of Terahertz Optoelectronics Ministry of Education, Capital Normal University, Beijing, 100048 China; 2 College of Physics and Electronics, Shandong Normal University, Jinan, 250014 China; 3 China Special Equipment Inspection and Research Institute, Beijing, 100029 China

## Abstract

A terahertz (THz) bottle beam is realized by adopting the combination of a Teflon axicon and a silicon lens. By using a THz imaging system with a focal-plane array, the vector characteristics of the THz bottle beam are coherently measured and detailedly analyzed, including the transverse (*E*_*x*_) and longitudinal (*E*_*z*_) components. The experimental phenomena vividly reveal the distribution characteristics and the formation origin of the THz optical barrier. A vectorial diffraction integral algorithm of a focusing optical system are utilized to exactly simulate the measured results. Besides, the features of the THz bottle beam are effectively tuned by varying the parameters of the Teflon axicon and the silicon lens. This work gives a full view to understand the evolution characteristics of the THz bottle beam and provide a solid experimental foundation for guiding the future applications of this type of THz beam.

## Introduction

Since the concept of an optical bottle beam was introduced by Arlt *et al*. in 2000^1^, this sort of special beam has attracted growing attentions due to its unique applications in particle trapping^2^ and simulated emission depletion (STED) fluorescence microscopy^3^. An optical bottle beam possesses an intensity null which are surrounded by an optical barrier in three dimensions so that it can be applied to realize the manipulation of low index particles or the enhancement of the longitudinal resolution in STED fluorescence microscopy. In the visible light range, the generation and modulation of an optical bottle beam have been widely investigated. In 2005, Wei *et al*. achieved the generation of an optical bottle beam by an apertured plane wave’s impinging through an axicon and a positive lens^4^. In 2013, Shvedov *et al*. utilized a uniaxial crystal to form a vector bottle beam and fulfilled the reconfiguration of the bottle beam by tuning the input beam polarization and the crystal parameters^5^. In 2014, Ye *et al*. proposed a scheme for generating a bottle-hollow beam using a binary phase mask and a focusing lens^6^. In 2017, Vella *et al*. proposed a generation method of an optical bottle field by placing a stress engineered optical window at the pupil of an aplanatic high numerical aperture focusing system^7^.

As a class of novel far-infrared optical inspection methods, the terahertz (THz) sensing and imaging technology has shown powerful application potentials in security inspection^8^, material identification^9^, non-invasive flaw detection^10^, and other numerous fields^11–13^ due to the properties of the THz radiation, such as the broad bandwidth, low photon energy, high penetration to non-metallic substances, and so on^14^. Currently, investigations of THz special beams have gradually become an important development orientation, because it is possible that distinctive features of these light beams are applied to broaden the scope of THz application. In 2012, a broadband Bessel THz beam was introduced into a THz scanning imaging system for enhancing the capability of obtaining depth information^15^. In 2015, the longitudinal component of a THz vector vortex beam was utilized to realize the linear acceleration of electrons^16^. In 2015, two separate orbital angular momentum channels were applied to achieve a sub-THz wireless communication link^17^. However, to our best knowledge, there is little investigation about an optical bottle beam in the THz frequency range in the previous reports.

In this work, the THz bottle beam is generated by employing the combination of a Teflon axicon and a silicon lens. The vector characteristics of the THz bottle beam are coherently measured, including the transverse (*E*_*x*_) and longitudinal (*E*_*z*_) electric fields. The evolution of the THz bottle beam is recorded and analyzed in detail by implementing a Z-scan measurement method. A vectorial diffraction algorithm is adopted to reproduce the features of the THz bottle beam. Besides, modulations of the THz bottle beam are fulfilled by varying the distance between the Teflon axicon and the silicon lens, the base angle of the Teflon axicon, and the focal length of the silicon lens. This work presents the vector characteristics and the basic modulation techniques for a THz bottle beam in detail.

## Experimental Design

**Figure 1:**
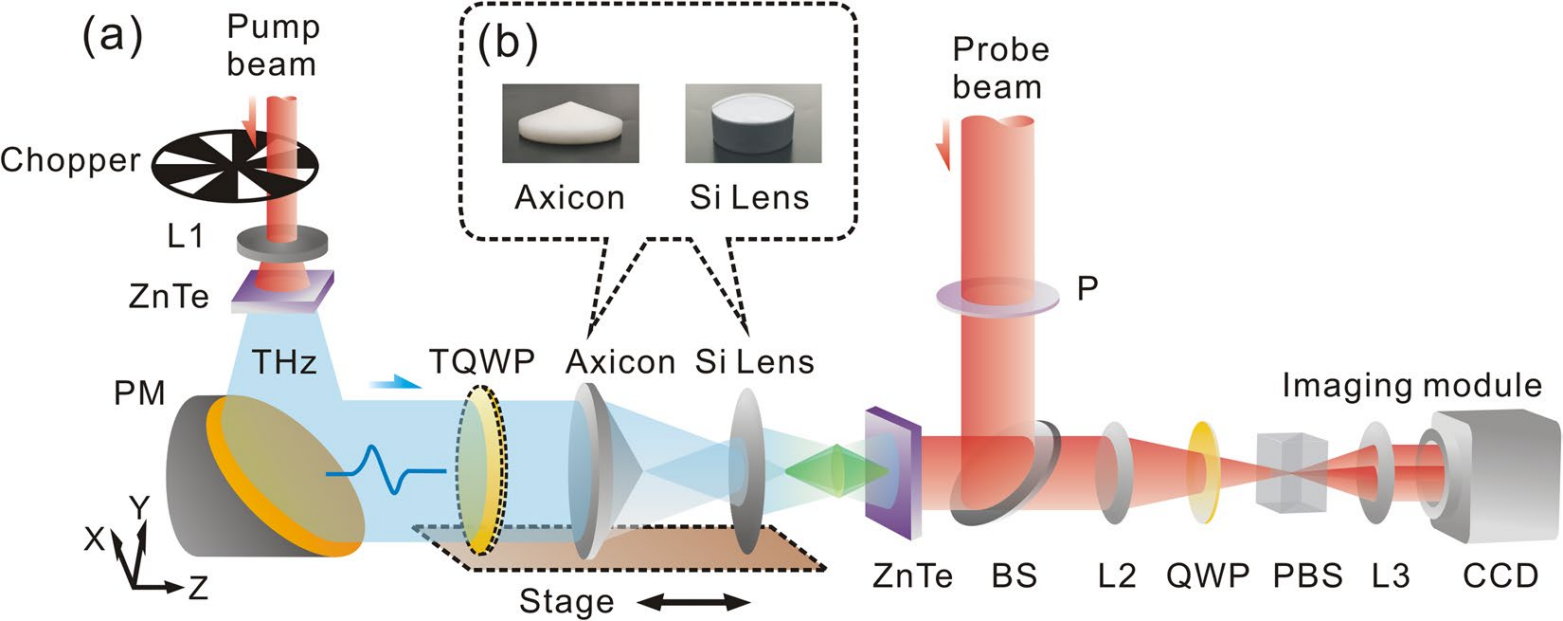
Experimental system and samples. **(a)** THz imaging system with a focal-plane array. **(b)** Pictures of a Teflon axicon and a silicon lens.

To acquire the vector characteristics of a THz bottle beam, a THz imaging system with a focal-plane array is used as the measurement platform. Figure 1a shows its schematic diagram. A THz quasi-plane wave with an x-linear polarization emerges through a Teflon axicon and a silicon lens to form a THz bottle beam, as shown in Fig. 1b. The transverse (*E*_*x*_) and longitudinal (*E*_*z*_) components of the THz bottle beam are coherently detected by applying the imaging system. To analyze the formation origin of the THz bottle beam, the positions of the Teflon axicon and the silicon lens are together successively adjusted for operating a Z-scan measurement. The diffraction process of the THz beam is recorded from z = −4.5 mm to z = 4.5 mm and the scanning step is set as 0.5 mm. It should be noted that the focal point of the silicon lens is viewed as the base point. In addition, a quartz THz quarter wave plate (TQWP, TYDEX Company, Russia) is also applied to vary the THz polarization.

## Results and Discussion

### Generation of a THz bottle beam

**Figure 2:**
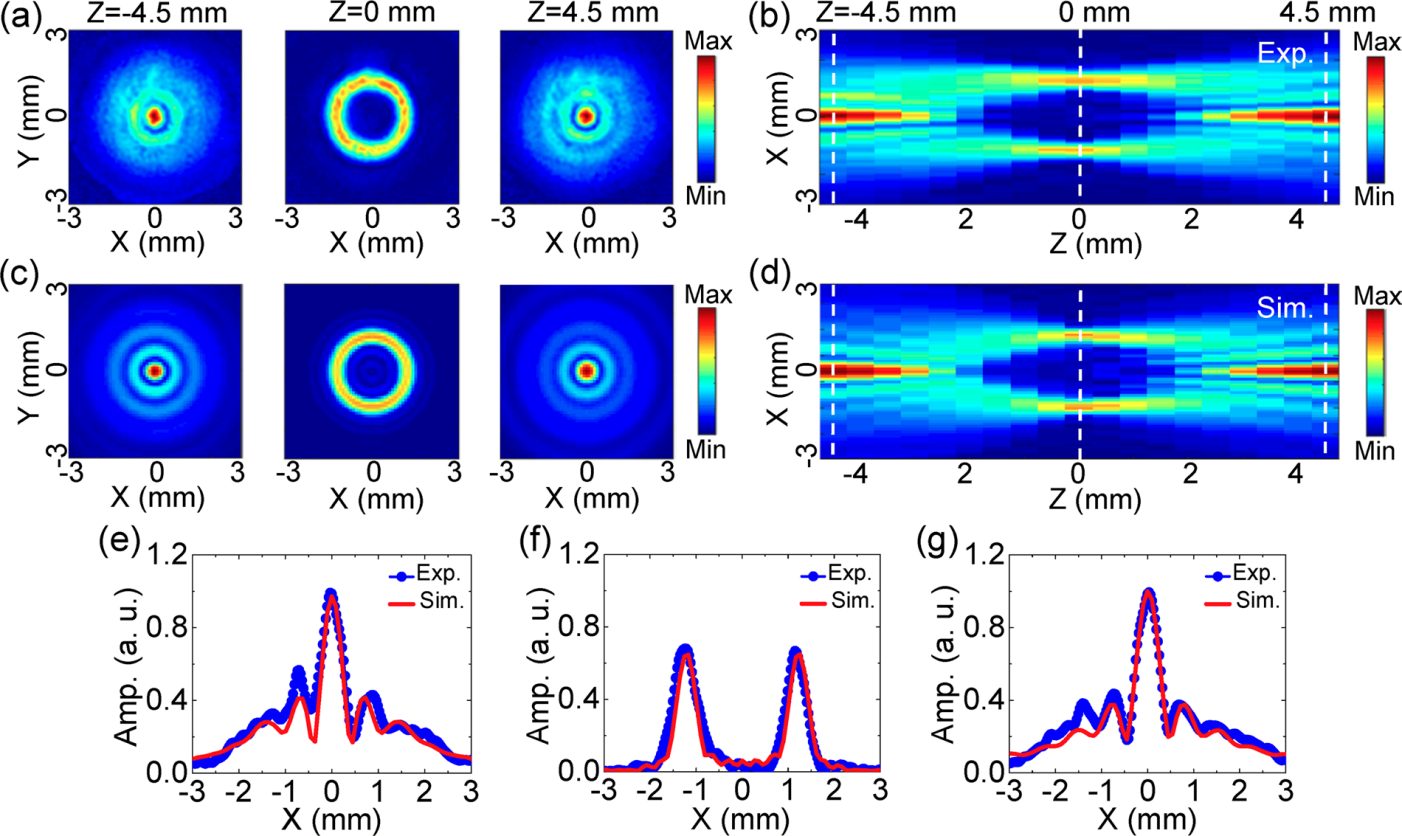
Amplitude image of *E*_*x*_ with 0.75 THz for the linearly polarized THz bottle beam. **(a)** Amplitude images of *E*_*x*_ on the x-y plane at z = −4.5 mm, 0 mm, 4.5 mm. **(b)** Longitudinal amplitude cross-section of *E*_*x*_ on the x-z plane. **(c)** and **(d)** gives the corresponding simulated results, including transverse amplitude images at z = −4.5 mm, 0 mm, 4.5 mm as well as the longitudinal amplitude pattern. **(e–g)** Plot the measured and calculated amplitude curves on the lines of z = −4.5 mm, 0 mm, 4.5 mm. Three white dashed lines are inserted in **(b,d)** to indicate their positions.

Firstly, a THz bottle beam with a x-linear polarization is formed by adopting a Teflon axicon with an base angle of 20° and a silicon lens with a focal length of 8 mm. The distance between the Teflon axicon and the silicon lens is about 35 mm. The 0.75 THz component is acquired by implementing the Fourier transformation. The amplitude images of *E*_*x*_ on the x-y plane at z = −4.5 mm, 0 mm, 4.5 mm are exhibited, as shown in Fig. 2a. Clearly, the THz field at z = −4.5 mm exhibits a circular central main spot with a diameter of 0.6 mm and two annular side-lobes with radiuses of 0.72 mm and 1.43 mm, which presents the distribution characteristics of a Bessel-like function. Here, the full widths at half maximum (FWHM) of the THz amplitude is viewed as the diameter of the main spot. The radius of the THz annular side-lobe is considered as the length between the maximum value and the central point. The amplitude image presents a doughnut pattern with a radius of 1.23 mm on the focal plane of the silicon lens (z = 0 mm), which possesses a central dark focus surrounded by a uniform optical barrier. After the THz beam passing through the focal plane, the amplitude image at z = 4.5 mm exhibits the Bessel-like distribution pattern once again, which are almost the same as the THz field at z = −4.5 mm. To observe the formation origin of the THz bottle beam, the cross-section of the THz amplitude on the x-z plane is extracted, as shown in Fig. 2b. Obviously, with the propagation of the THz beam, the central peak intensity of the THz field gradually attenuates and the dark focus appears until the sharpest ring of the THz beam is formed from z = −4.5 mm to 0 mm. After passing through the position of z = 0 mm, the reverse diffraction process is generated. Namely, the edge of the THz light ring progressively becomes ambiguous and the central dark focus starts to shrink. Finally, the THz intensity concentrates into the center and become a peak again. The evolution properties of the THz beam are consistent with those of a typical optical bottle beam^1^.

According to ref.^4^, the radius of the sharpest THz light ring can be expressed as

1 $$R=f\,\tan (\arcsin (n\,\sin \,\alpha )\,-\alpha ),$$

where *α* and *n* is the base angle and the refractive index of the Teflon axicon, *f* is the focal length of the silicon lens. Herein, these parameters are *α* = 20°, *n* = 1.426^18^, *f* = 8 mm, respectively. The calculated *R* equals 1.29 mm, which is generally in agreement with the measured value. In addition, in the interior of the THz bottle beam, the longitudinal distance of the central dark region along the z direction can be evaluated as

2 $${\rm{\Delta }}z=\frac{{z}_{0}\,f}{{z}_{0}-f}-\frac{f-\frac{{z}_{0}\,f}{{Z}_{\max }}}{1+\frac{f}{{Z}_{\max }}-\frac{{z}_{0}}{{Z}_{\max }}},$$

where *z*_0_ is the distance between the Teflon axicon and the silicon lens, ${Z}_{\max }={\omega }_{{\rm{0}}}/\,\tan [\arcsin (n\,\sin \,\alpha )-\,\alpha ]$ is the diffraction-free range of the THz Bessel beam formed by the Teflon axicon, *ω*_0_ is the radius of the incoming THz beam. Herein, these parameters are *z*_0_ = 35 mm, *ω*_0_ = 7 mm, *Z*_max_ = 43 mm. The measured and calculated Δ*z* are 6.0 mm and 6.30 mm, which are basically consistent with each other. Here, when the forward and backward optical barriers on the negative and positive positions of the z axis reach the same intensities as the THz light ring on the focal plane, the distance between them is defined as the longitudinal distance Δ*z* of the THz bottle beam.

To simulate these measured phenomena, the vectorial diffraction integral algorithm of a focusing optical system is adopted under the Debye approximation^19^. Close to the focal spot, the vector electric field of the converging THz beam can be described as

3 $${\boldsymbol{E}}(\rho ,\phi ,z)=-\,j\frac{f}{\lambda }{\int }_{0}^{\beta }{\int }_{0}^{2\pi }T(\theta ,\varphi ){\boldsymbol{P}}(\theta ,\varphi )\exp [jk(\rho \,\sin \,\theta \,\cos (\varphi -\phi )+z\,\cos \,\theta )]\sin \,\theta d\theta d\varphi ,$$

where *λ* is the wavelength of the incoming THz wave, *k* is the wave number in vacuum, *β* is the maximum convergence angle of the THz beam after the silicon lens, $(\rho ,\phi ,z)$ are the cylindrical coordinates of the focal area, $(\theta ,\varphi )$ are the spherical angular coordinates of the output pupil of the silicon lens, $T(\theta ,\varphi )$ is the pupil apodization function of the silicon lens and can be expressed as $T(\theta ,\varphi )=\sqrt{\cos \,\theta }$, ${\boldsymbol{P}}(\theta ,\varphi )$ is the polarization matrix of the silicon lens. ${\boldsymbol{P}}(\theta ,\varphi )$ has the following expression

4 $${\boldsymbol{P}}(\theta ,\varphi )=[\begin{array}{ccc}1+{\cos }^{2}\varphi (\cos \,\theta -1) & \sin \,\varphi \,\cos \,\varphi (\cos \,\theta -1) & \cos \,\varphi \,\sin \,\theta \\ \sin \,\varphi \,\cos \,\varphi (\cos \,\theta -1) & 1+{\sin }^{2}\varphi (\cos \,\theta -1) & \sin \,\varphi \,\sin \,\theta \\ -\sin \,\theta \,\cos \,\varphi  & -\sin \,\theta \,\sin \,\varphi  & \cos \,\theta \end{array}]\times [\begin{array}{c}{i}_{x}\\ {i}_{y}\\ {i}_{z}\end{array}],$$

where *i*_*x*_, *i*_*y*_, and *i*_*z*_ are the polarization functions for x-, y- and z-components on the entrance pupil of the silicon lens. On the incidence plane of the silicon lens, the THz field refracted by the Teflon axicon forms an x-linearly polarized Bessel beam, so *i*_*x*_, *i*_*y*_, and *i*_*z*_ can be written as^20^

5 $$\begin{array}{rcl}{i}_{x}(\rho ^{\prime} ,\varphi ) & = & \frac{-j{z}_{0}\pi \exp (jkr)}{\lambda {r}^{2}}\\  &  & \times {\int }_{0}^{l}d{\rho }_{0}\exp (\mu ){\rho }_{0}[({t}_{p}\,\cos \,\sigma +{t}_{s})\\  &  & \times {J}_{0}(\eta )-({t}_{p}\,\cos \,\sigma -{t}_{s}){J}_{2}(\eta )\cos \,2\varphi ],\end{array}$$

6 $${i}_{y}(\rho ^{\prime} ,\varphi )=0,$$

7 $$\begin{array}{rcl}{i}_{z}(\rho ^{\prime} ,\varphi ) & = & \frac{j\pi \exp (jkr)\cos \,\varphi }{\lambda {r}^{2}}\\  &  & \times {\int }_{0}^{l}d{\rho }_{0}\exp (\mu ){\rho }_{0}[\begin{array}{c}({t}_{p}\,\cos \,\sigma +{t}_{s})\rho ^{\prime} {J}_{0}(\eta )\\ -2j{t}_{p}{\rho }_{0}\,\cos \,\sigma {J}_{1}(\eta )-({t}_{p}\,\cos \,\sigma -{t}_{s})\rho ^{\prime} {J}_{2}(\eta )\end{array}],\end{array}$$

where $(\rho ^{\prime} ,\varphi )$ are the polar coordinates on the entrance pupil of the silicon lens and $\rho ^{\prime}$ can be expressed as $\rho ^{\prime} =f\,\sin \,\theta$, *t*_*p*_ and *t*_*s*_ are the Fresnel transmission ratios for x- and y-polarization components, *l* is the aperture radius of the Teflon axicon, *σ* is the deflection angle between the z axis and the out-going THz beam from the Teflon axicon and can be written as $\sigma =\arcsin (n\,\sin \,\alpha )-\alpha$. Here, $r=\sqrt{{\rho ^{\prime} }^{2}+{z}_{0}^{2}}$ and ${\rho }_{0}=\sqrt{{x}_{0}^{2}+{y}_{0}^{2}}$, where $({x}_{0},{y}_{0})$ are the rectangular coordinates on the incidence plane of the Teflon axicon. ${J}_{0}(\eta )$, ${J}_{1}(\eta )$ and ${J}_{2}(\eta )$ are the Bessel functions of the first kind with the topological charges of 0, 1, 2. $\eta$ and $\mu$ can be written as

8 $$\eta =-\,\frac{k\rho ^{\prime} {\rho }_{0}}{r},$$

9 $$\mu =[-\frac{{\rho }_{0}^{2}}{{\omega }_{0}^{2}}+jk\frac{{\rho }_{0}^{2}}{2r}-jk{\rho }_{0}(n-1)\tan \,\alpha ].$$

Figure 2c shows the simulated amplitude distributions of *E*_*x*_ at z = −4.5 mm, 0 mm, 4.5 mm for the THz bottle beam, which are consistent with the measured phenomena. On the longitudinal cross-section, the simulated THz amplitude pattern is also extracted, as shown in Fig. 2d. The simulated result exactly presents the evolution of the THz bottle beam as the experimental one. To further check the accuracy of the simulated result, the measured and calculated amplitude curves at z = −4.5 mm, 0 mm, 4.5 mm are extracted from Fig. 2b,d. Three white dashed lines are inserted into Fig. 2b,d to indicate their positions. Figure 2e–g exhibit the comparison of the measured and calculated results, which show that the maximum divergence does not exceed 15%.

**Figure 3:**
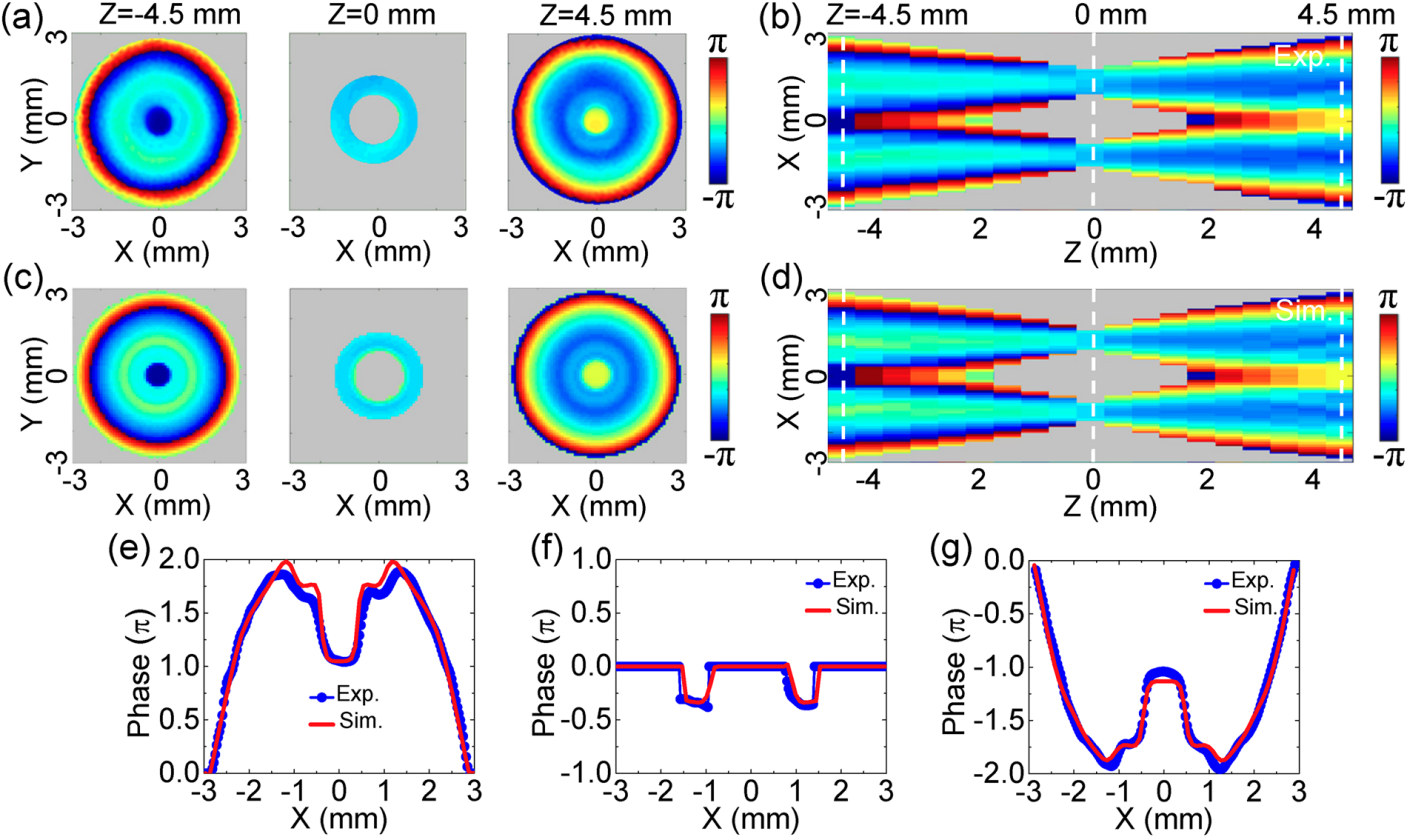
Phase pattern of *E*_*x*_ with 0.75 THz for the linearly polarized THz bottle beam. **(a)** Transverse wrapped phase images of *E*_*x*_ at z = −4.5 mm, 0 mm, 4.5 mm. **(b)** Longitudinal wrapped phase cross-section of *E*_*x*_. **(c,d)** Show the simulated transverse and longitudinal phase patterns. **(e–g)** show the measured and calculated unwrapped phase curves along the x axis at z = −4.5 mm, 0 mm, 4.5 mm. Three white dashed lines in **(b,d)** indicate their positions.

Because the THz field is coherently detected by the imaging system, the THz phase information can be directly obtained. To more intuitively observe the features of the THz bottle beam, the wrapped phase patterns of *E*_*x*_ with 0.75 THz at z = −4.5 mm, 0 mm, 4.5 mm are acquired, as shown in Fig. 3a. Here, the color of a pixel is set as gray and the corresponding phase value is fixed as 0 when the amplitude is less than 10% of the maximum value on the pixel. The aim of the operation is to filter the phase noise on each phase image. Obviously, all of the three phase patterns present axial symmetric distributions, because both of the Teflon axicon and the silicon lens induce axial symmetric phase modulations. At z = −4.5 mm, the phase pattern shows a convex shape from *ρ* = 0.27 mm to 3.0 mm. Meanwhile, the phase pattern exhibits a concave shape along the radial direction at z = 4.5 mm. At z = 0 mm, the phase value remains almost invariable near the annular region of *ρ* = 1.23 mm. For clarity, the phase curves along the lines of y = 0 mm are extracted from the three phase images and are unwrapped, as shown in Fig. 3e–g. These phenomena manifest that the THz Bessel beam induced by the Teflon axicon undergoes converging and diverging processes to ultimately form the THz bottle beam after passing through the silicon lens. Figure 3b shows the longitudinal cross-section of the *E*_*x*_ wrapped phase, which more vividly presents the propagation process of the THz beam. It can be said that the Bessel-like THz field is due to the interference effect of converging THz beams at z = −4.5 mm or diverging THz beams at z = 4.5 mm, respectively^4^. At z = 0 mm, the ring-shaped THz field is composed by a series of focal spots of converging THz beams. According to ref.^21^, the annular THz intensity can be considered as the Fourier transformation of the THz Bessel beam. By utilizing Eqs (3–9), the phase pattern of *E*_*x*_ is also simulated for the THz bottle beam. Figure 3c shows the simulated wrapped phase images at z = −4.5 mm, 0 mm, 4.5 mm and Fig. 3d gives the corresponding longitudinal phase cross-section, which are in accordance with the measured phenomena. The calculated unwrapped phase curves at z = −4.5 mm, 0 mm 4.5 mm are also extracted along the lines of y = 0 mm from Fig. 3c and are compared with the measured results, as shown in Fig. 3e–g. The measured and calculated results are in accordance with each other. The positions of the measured and calculated phase curves are also indicated by the three white dashed lines in Fig. 3b,d. It can be seen that the formation origin of the THz optical barrier can be easily understand by analyzing the complex field information of the THz bottle beam.

### Vector characteristics of a THz bottle beam

**Figure 4:**
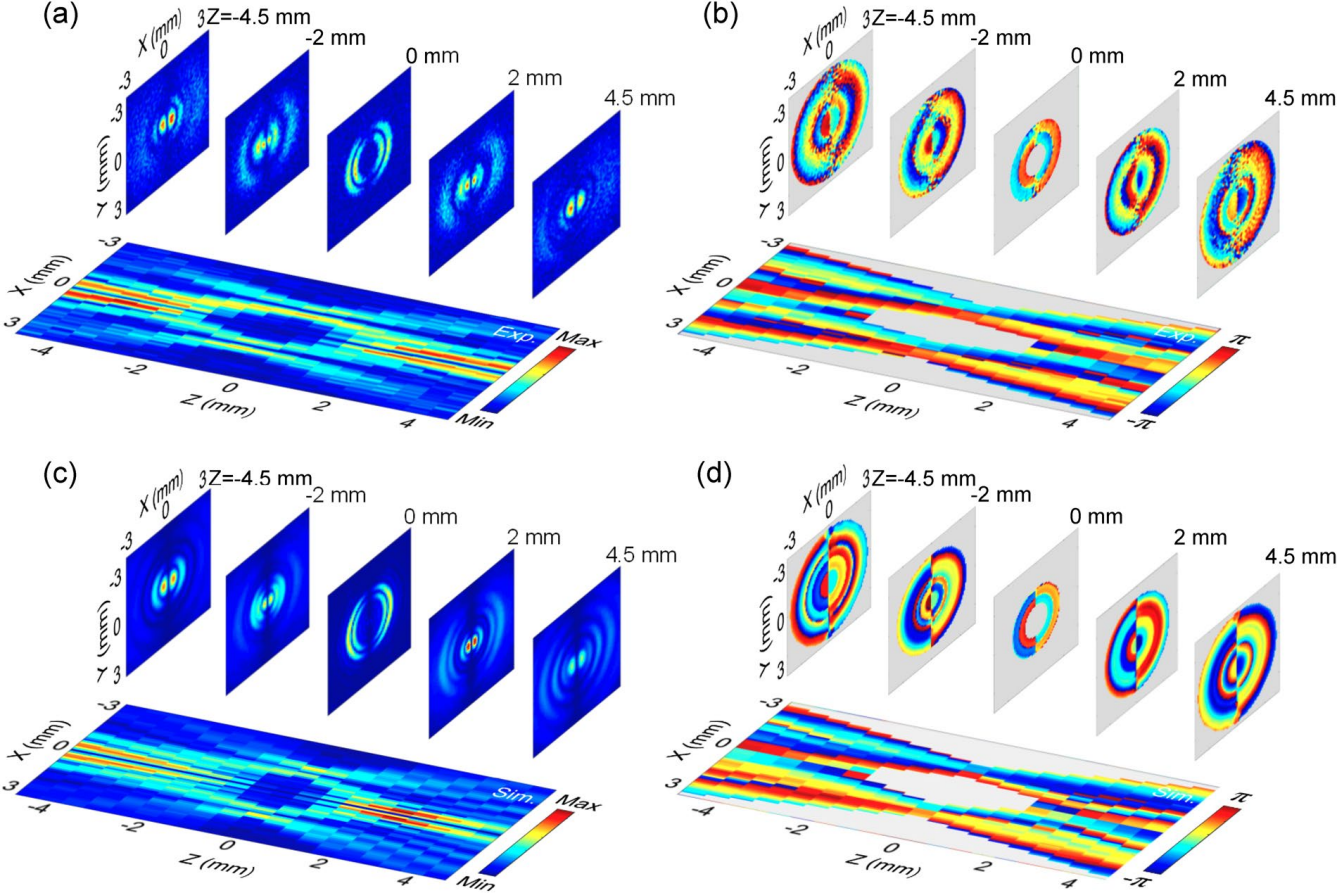
Complex field pattern of *E*_*z*_ with 0.75 THz for the linearly polarized THz bottle beam. **(a,b)** show the *E*_*z*_ amplitude and wrapped phase images on the x-y plane at z = −4.5 mm, −2 mm, 0 mm, 2 mm, 4.5 mm. The *E*_*z*_ amplitude and phase cross-sections on the x-z plane are also extracted and presented. **(c,d)** give the corresponding simulated *E*_*z*_ amplitude and phase patterns.

Taking advantage of the vector characterization function of the THz imaging system, the diffraction properties of the *E*_*z*_ component are measured and analyzed for the THz bottle beam. The identical x-linearly polarized THz bottle beam is formed by applying the Teflon axicon with *α* = 20° as well as the silicon lens with *f* = 8 mm and its *E*_*z*_ component with 0.75 THz is extracted. By fulfilling the Z-scan scheme, the evolution of *E*_*z*_ is coherently recorded. Figure 4a exhibits the amplitude distributions of *E*_*z*_ on the x-y plane at z = −4.5 mm, −2 mm, 0 mm, 2 mm, 4.5 mm and the amplitude cross-section on the x-z plane. The *E*_*z*_ component shows interesting distribution characteristics. At z = −4.5 mm, the *E*_*z*_ component possesses two off-axis main-lobes at x = ±0.33 mm as well as several crescent side-lobes, which presents a bilateral symmetric amplitude distribution. The amplitude maximum of *E*_*z*_ is approximately 25% of *E*_*x*_. Around the optical axis, there is a zero-amplitude zone. With increasing the propagation distance, the structure of the *E*_*z*_ component starts to become ambiguous. At z = −2 mm, the off-axis main-lobes as well as the inner side-lobes have mixed together and the outer side-lobes show a converging trend to the inside. When the THz beam propagates to z = 0 mm, the *E*_*z*_ component exhibits two pairs of crescent amplitude patterns, which shows a mirror symmetric configuration. Each pair of the amplitude patterns separately locates on the left and right sides of x = ±1.23 mm. After passing through the focal plane, the THz beam undergoes an inverse evolution process. The longitudinal amplitude pattern more clearly manifests the variation of *E*_*z*_ during the formation of the THz bottle beam. To explain the phenomena, the transverse wave vectors are induced and *E*_*z*_ is generated in the focusing region of the THz beam. Furthermore, *E*_*z*_ should present the intensity distributions on the two sides of the corresponding focal spot of *E*_*x*_ for the linearly polarized focusing THz beam^22^. At z = −4.5 mm and 4.5 mm, *E*_*x*_ manifests the distribution pattern of a Bessel-like beam, including a circular central peak and ring-shaped side-lobes. Therefore, the corresponding *E*_*z*_ component shows off-axis main-lobes and crescent side-lobes, which are in agreement with our previous report^18^. On the focal plane (z = 0 mm), the focal spot of *E*_*x*_ locates on the position of the THz light ring, so the *E*_*z*_ intensities appear on the two sides of the THz light ring. In addition, there is no any *E*_*z*_ component on the y-z plane because the THz polarization is along the x direction^22^. Figure 4b show the transverse wrapped phase patterns of *E*_*z*_ at z = −4.5 mm, −2 mm, 0 mm, 2 mm, 4.5 mm and the longitudinal phase cross-section. The phase of *E*_*z*_ manifests a complicated distribution which consists of several semi-ring regions. These semi-ring regions exhibit a mirror symmetric distribution with respect to the y axis. Besides, it is not difficult to find that there is always a phase jump of π between the corresponding left and right semi-ring regions. It means that the propagation directions of the *E*_*z*_ components are always opposite on the two sides of the y-z plane. By utilizing Eqs (3–9), the simulated amplitude and phase images of *E*_*z*_ are acquired. Figure 4c,d show the calculated results at z = −4.5 mm, −2 mm, 0 mm, 2 mm, 4.5 mm and the longitudinal amplitude and phase cross-sections, which are in accordance with the measured results. Besides, it should be also noted that there is not a tight-focusing process during the formation of the THz bottle beam, so the polarization of the THz field is not influenced and the *E*_*y*_ component can be negligible^23^.

**Figure 5:**
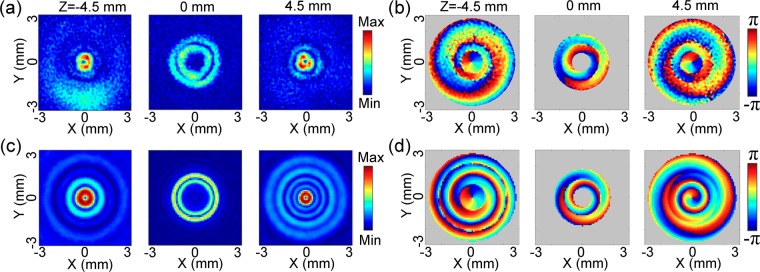
Complex field pattern of *E*_*z*_ for the THz bottle beam with a left circular polarization. **(a,b)** Present the amplitude and wrapped phase patterns of *E*_*z*_ at z = −4.5 mm, 0 mm, 4.5 mm on the x-y plane. **(c,d)** Give the simulated results.

In the work, the *E*_*z*_ component is also observed for a THz bottle beam with a circular polarization. By inserting the quartz TQWP into the path of the incoming THz beam, the left circularly polarized THz bottle beam is generated and the complex field of its *E*_*z*_ component is coherently recorded. Figure 5a gives the amplitude images of *E*_*z*_ on the x-y plane at z = −4.5 mm, 0 mm, 4.5 mm, respectively. The *E*_*z*_ component shows similar amplitude distributions at z = −4.5 mm and 4.5 mm, including a central doughnut-shaped intensity and some weak annular side-lobes. On the focal plane (z = 0 mm), the *E*_*z*_ component obviously exhibits two concentric light rings. Figure 5b shows the wrapped phase patterns at z = −4.5 mm, 0 mm, 4.5 mm, which clearly present vortex modalities. Their phase values linearly increase in a clockwise sense. Besides, the twist sense of the *E*_*z*_ phase exhibits a reverse after propagating through the focal plane. Actually, the phase characteristics are analogous to the *E*_*z*_ phase properties of a converging circularly polarized THz Gaussian beam^22^. When the polarization of a focusing light beam is adjusted as the circular polarization, both *E*_*z*_ components are excited on the x-z and y-z planes. Simultaneously, there is a phase delay of π/2 between them. Ultimately, their interference effect forms the annular amplitude and the spiral phase. According to our previous reports^18^, a general law has been summarized for the *E*_*z*_ component of a converging light beam. Namely, when a light beam emerges through a set of axial symmetrical wave front modulators, *E*_*z*_ with a linear or a circular polarization presents a double-lobe characteristics or a vortex mode. Obviously, the Teflon axicon and the silicon lens possess the amplitude and phase modulations with an axial symmetry to the incoming THz beam, so the formed THz bottle beam conforms to the general law. The complex field of *E*_*z*_ with a circular polarization can be viewed as the linear superposition of *E*_*xz*_ and *E*_*yz*_. Herein, *E*_*xz*_ and *E*_*yz*_ are longitudinal components of two THz bottle beams with perpendicular linear polarizations. Accordingly, *E*_*z*_ with the left circular polarization can be written as

10 $${E}_{z}={E}_{xz}+{E}_{yz}\exp (-j\frac{\pi }{2}).$$

The calculated amplitude and phase images of *E*_*z*_ at z = −4.5 mm, 0 mm, 4.5 mm are given in Fig. 5c,d, which are in accordance with the measured phenomena.

### Performance tuning of a THz bottle beam

**Figure 6:**
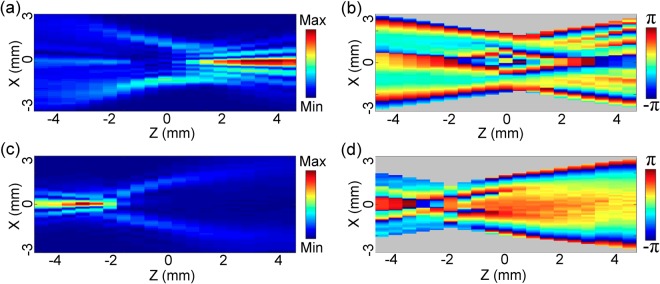
Modulation of the THz bottle beam by varying the distance between the Teflon axicon and the silicon lens. **(a,b)** Present the longitudinal *E*_*x*_ amplitude and wrapped phase cross-sections when the distance is adjusted as 56 mm. **(c,d)** Show the *E*_*x*_ amplitude and phase patterns on the x-z plane when the distance is set as 6 mm.

If the parameters of the Teflon axicon and the silicon lens are tuned, the characteristics of the formed THz bottle beam can be effectively modulated. Firstly, the distance *z*_0_ between the Teflon axicon with *α* = 20° and the silicon lens with *f* = 8 mm are adjusted to observe the variation of the THz bottle beam. Figure 6a and c separately give the longitudinal amplitude patterns of *E*_*x*_ with 0.75 THz when *z*_0_ are set as 56 mm and 6 mm. Interestingly, the forward optical barrier (the central peak at z = −4.5 mm) of the THz bottle beam is diminished when *z*_0_ is equal to 56 mm. Meanwhile, the backward optical barrier (the central peak at z = 4.5 mm) is attenuated if *z*_0_ is adjusted to 6 mm. To explain these phenomena, the THz beam refracted by the Teflon axicon forms a series of THz plane waves propagating along different directions, which emerge through the silicon lens and generate the THz bottle beam if *f* < *z*_0_ < *Z*_max_. When the distance *z*_0_ is too large, these converging THz beams after passing through the silicon lens cannot overlap with each other so that the forward optical barrier disappears. In the same way, when the distance *z*_0_ is too small, these diverging THz beams after propagating through the focal plane cannot overlap with each other, so the backward optical barrier vanishes. Figure 6b,d exhibit the longitudinal wrapped phase cross-sections of *E*_*x*_ with *z*_0_ = 56 mm and 6 mm, which also clearly manifest these processes. When *z*_0_ is set as 56 mm or 6 mm, the phase pattern only expresses the obvious interference around z = 4.5 mm or −4.5 mm, respectively. Therefore, it can be concluded that the opening direction of the THz optical barrier can be readily controlled by adjusting the distance between the Teflon axicon and the silicon lens.

**Figure 7:**
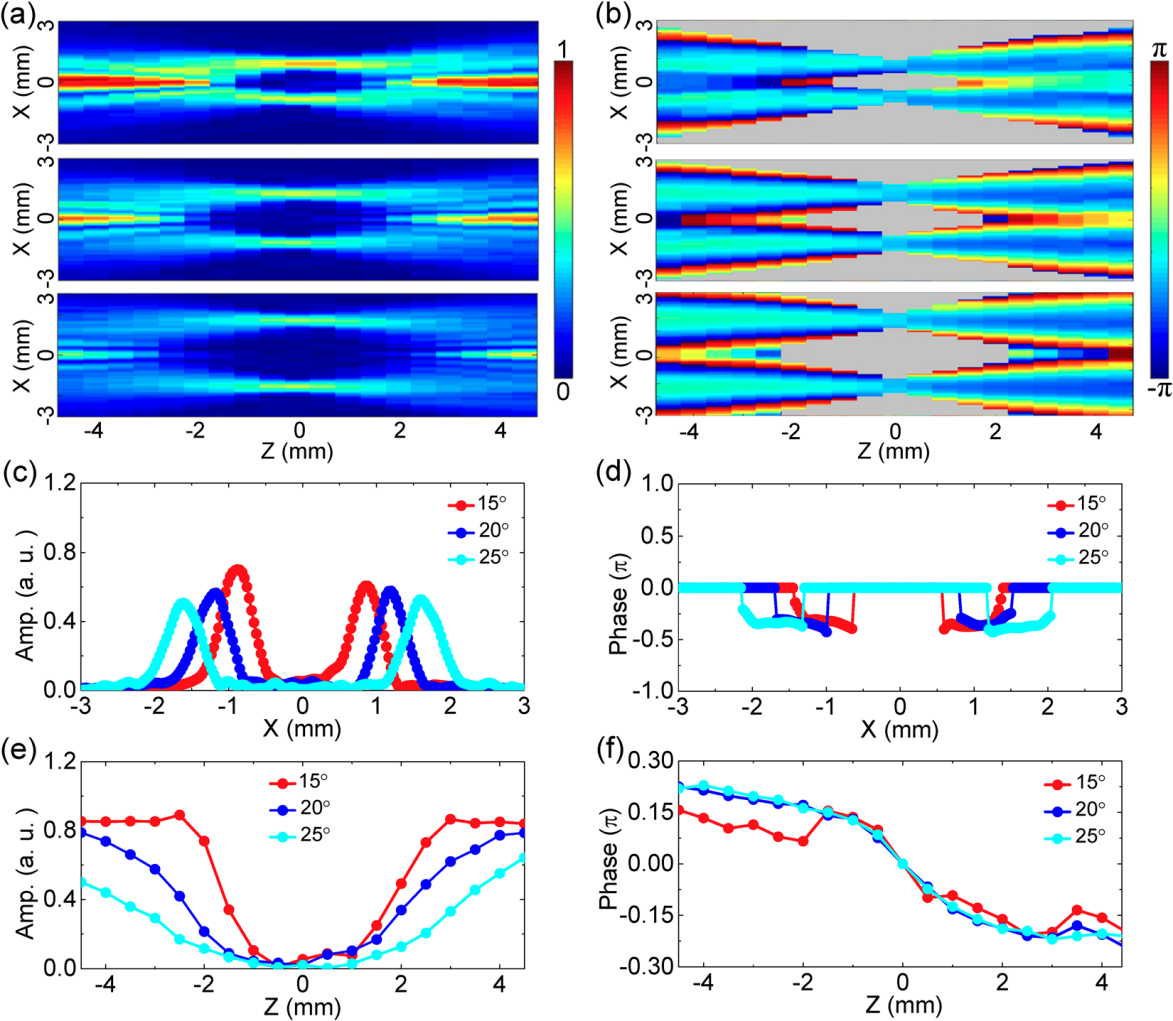
Variance of the THz bottle beam with adjusting the base angle of the Teflon axicon. **(a,b)** Present the longitudinal *E*_*x*_ amplitude and wrapped phase images when the silicon lens with the focal length of *f* = 8 mm and the Teflon axicons with the base angles of *α* = 15°, 20°, 25° are picked to form the THz bottle beams. **(c,e)** Give the amplitude curves with *α* = 15°, 20°, 25°. These curves are extracted along the lines of z = 0 mm and x = 0 mm in **(a)**. **(d)** Shows the phase curves with *α* = 15°, 20°, 25°, which are extracted along the lines of z = 0 in **(b)**. **(f)** Exhibits the longitudinal phase curves which are extracted from **(b)** on the amplitude maximal positions with *α* = 15°, 20°, 25°.

The properties of the THz bottle beam are also sensitively dependent on the base angle of the Teflon axicon. To check this point, the Teflon axicons with *α* = 15°, 20°, 25° and the silicon lens with *f* = 8 mm are selected to realize discrepant THz bottle beams. The Z-scan characterization method is operated to record the propagations of these THz bottle beams and their divergences are compared in detail. To acquire the complete optical barriers of these THz beams, the distances between the Teflon axicons with *α* = 15°, 20°, 25° and the silicon lens are carefully adjusted to 44 mm, 35 mm, 29 mm, respectively. Figure 7a exhibits the longitudinal amplitude patterns of *E*_*x*_ with *α* = 15°, 20°, 25° at 0.75 THz. For comparison, these images are normalized with respect to the amplitude maximum with *α* = 15°. Clearly, with increasing the base angle of the Teflon axicon, the intensity of the optical barrier is gradually attenuated and both of the transverse size and the longitudinal distance of the THz bottle beam are progressively magnified. To quantitively analyze characteristics differences between these THz beams, the amplitude curves with *α* = 15°, 20°, 25° are separately extracted from Fig. 7a along the lines of z = 0 mm and x = 0 mm, as shown in Fig. 7c,e. The measured results show that the radiuses of these THz annular amplitudes are 0.89 mm, 1.23 mm, 1.68 mm and the longitudinal distances of these THz bottle beams are 4.5 mm, 6.0 mm, 8.5 mm, respectively. According to Eqs (1–2) the calculated *R* and Δ*z* are separately 0.93 mm, 1.29 mm, 1.70 mm as well as 4.45 mm, 6.30 mm, 8.49 mm, which are generally consistent with the measured results. To understand the phenomena, a Teflon axicon with a larger base angle can induce a bigger deflection angle between the z axis and the refracted THz beam. When these refracted THz beams emerge through the silicon lens, the superposition region of the converging or diverging THz beams is shrunk before or after the focal plane so that the intensity of the optical barrier is weakened and the central dark region of the THz bottle beam is enlarged. To further confirm this point, the longitudinal wrapped phase cross-sections of the *E*_*x*_ components with *α* = 15°, 20°, 25° are acquired, as shown in Fig. 7b. It can be seen that the spatial interval between the converging THz beams gradually increases at z = −4.5 mm and the superposition region between the diverging THz beams continuously reduces at z = 4.5 mm with increasing *α*. Specially, the part regions of the converging and diverging THz beams with *α* = 25° have gone beyond the view field of the imaging system. The reason may also results in the intensity attenuation of the optical barrier to a certain degree. The phase curves with *α* = 15°, 20°, 25° are extracted from Fig. 7b along the lines of z = 0 mm, as shown in Fig. 7d. The measured results manifest that the positions of the phase flat regions with *α* = 15°, 20°, 25° are approximately x = ±0.89 mm, ±1.23 mm, ±1.68 mm. In these phase patterns, the longitudinal phase curves are obtained on the corresponding amplitude maximal positions, as shown in Fig. 7f. For clarity, the original values of these curves at z = 0 mm are fixed as 0. Obviously, the phase evolutions with *α* = 15°, 20°, 25° manifest the almost same Gouy phase shifts, which are the phase differences of 0.5π between z = −4.5 mm and 4.5 mm. The reason can be explained that all of THz beams refracted by the Teflon axicons with various base angles are focused by the silicon lens with *f* = 8 mm. After the silicon lens, these out-going THz beams with different *α* undergo the similar converging process, so their longitudinal phase evolutions are almost identical. In addition, it can be seen that the Gouy phase shift of a bottle beam is only half of that of a converging Gaussian beam^24^. To understand the phenomenon, a transmitted THz Bessel beam from the silicon lens is only one-dimensionally focused on each radial cross-section along the propagation direction, so that a THz light ring on the focal plane and a Gouy phase shift of 0.5π are formed. Besides, there are some oscillations on the phase curve with *α* = 15°, which may be caused by the more significant interferences of the converging or diverging THz beams with a smaller *α*.

**Figure 8:**
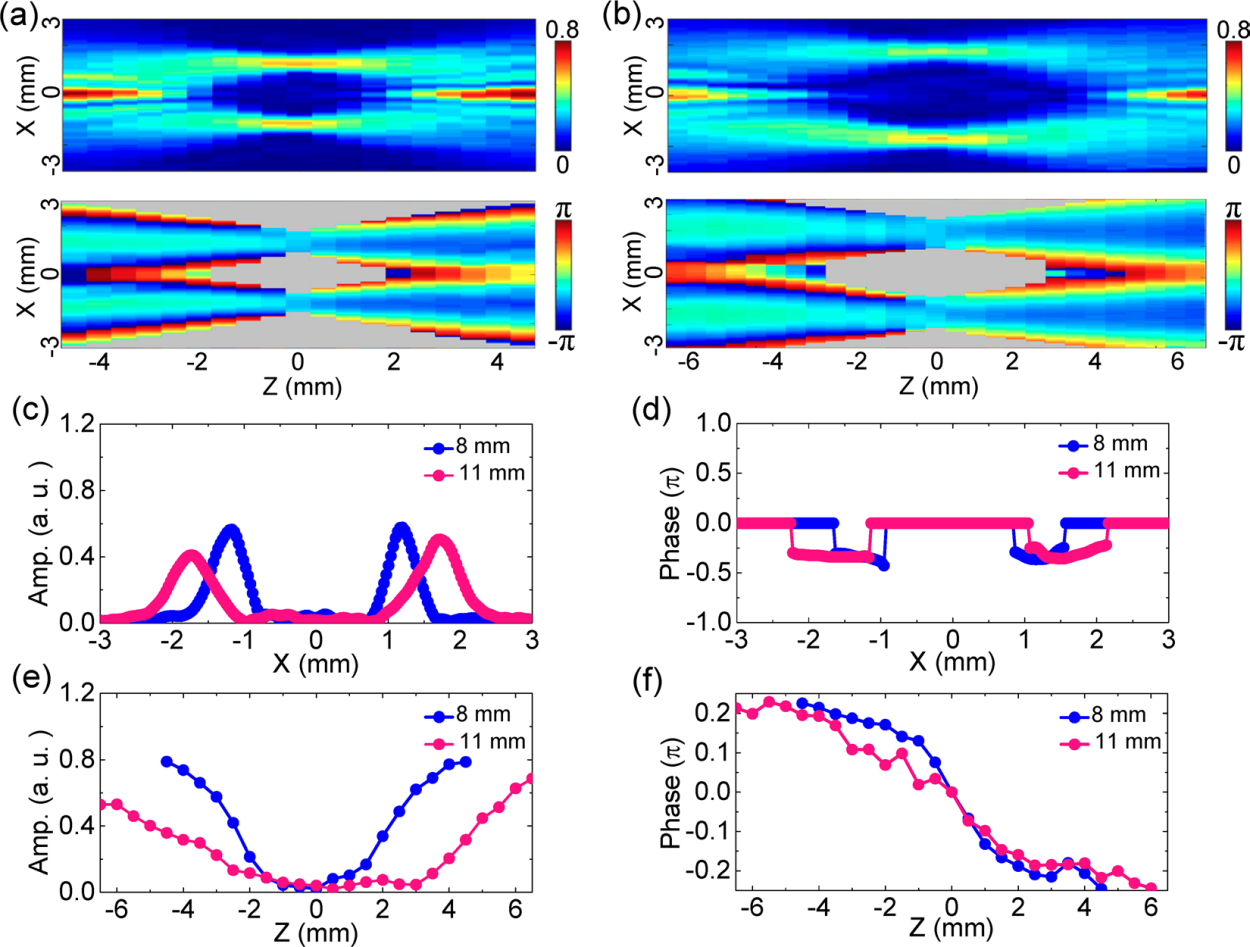
Influence of the focal length of the silicon lens to the THz bottle beam. **(a)** Presents the *E*_*x*_ amplitude and wrapped phase cross-sections with *α* = 20° and *f* = 8 mm on the x-z plane. **(b)** Shows the longitudinal *E*_*x*_ amplitude and wrapped phase patterns with *α* = 20° and *f* = 11 mm. **(c,e)** Give the amplitude curves with *f* = 8 mm and 11 mm along the lines of z = 0 mm and x = 0 mm. **(d)** Shows the transverse phase curves with *f* = 8 mm and 11 mm along the lines of z = 0 mm. **(f)** Plot the longitudinal phase curves with *f* = 8 mm and 11 mm on the corresponding amplitude maximal positions.

Finally, the influence of the focal length of the silicon lens to the THz bottle beam is also investigated. The Teflon axicon with *α* = 20° and the silicon lens with *f* = 11 mm are picked to generate the THz bottle beam. The distance between them is carefully adjusted to 39 mm for ensuring a complete optical barrier. The Z-scan characterization method is operated to reconstruct the propagation of the THz bottle beam. Herein, the scan range is from −6.5 mm to 6.5 mm and the scanning step is 0.5 mm. Figure 8b exhibits the longitudinal amplitude and wrapped phase cross-sections of *E*_*x*_ with 0.75 THz. For comparison, the corresponding longitudinal *E*_*x*_ amplitude and wrapped phase patterns of the THz bottle beam with *α* = 20° and *f* = 8 mm are also given in Fig. 8a. Comparing these amplitude images, the intensity of the optical barrier is attenuated and the scale of the central dark focus is magnified for the THz bottle beam formed by using a silicon lens with a longer focal length. From these phase images, it can be seen that the phase variation tendency of the THz bottle beam is slower with increasing *f*. The reason inducing these phenomena is easy to understand. When a silicon lens with a longer focal length is adopted, the THz beam experiences a smoother focusing process so that the weaker optical barrier is formed. To quantitively observe the variation of the THz bottle beam, the amplitude curves are acquired along the transverse (z = 0 mm) and longitudinal (x = 0 mm) directions, as shown in Fig. 8c,e. The radiuses of the THz light rings with *f* = 8 mm and 11 mm are 1.23 mm and 1.73 mm on their focal planes, respectively. The longitudinal distances of these central dark regions are 6.0 mm and 12.0 mm for the THz bottle beams with *f* = 8 mm and 11 mm. Utilizing Eqs (1–2), the calculated *R* and Δ*z* with *f* = 8 mm and 11 mm are 1.29 mm, 6.30 mm as well as 1.78 mm, 12.24 mm, which are generally in accordance with the measured results. The transverse phase curves with *f* = 8 mm and 11 mm along the lines of z = 0 mm are obtained and shown in Fig. 8d. It can be observed that the positions of the phase flat regions with *f* = 8 mm and 11 mm are approximately x = ±1.23 mm and ±1.73 mm. Besides, the phase flat region with a larger *f* obviously exhibits a broader width. The phase flat regions with *f* = 8 mm and 11 mm separately possess the lateral scales of 0.67 mm and 1.06 mm. On the corresponding amplitude maximal positions, the longitudinal phase curves with *f* = 8 mm and 11 mm are also acquired and compared. In Fig. 8f, it can be observed that the phase difference with *f* = 11 mm between z = −6.5 mm and 6.5 mm also reaches 0.5π and shows a smoother variation tendency than the Gouy phase shift with *f* = 8 mm. The phenomena manifest that when a silicon lens with a longer focal length is chosen, the focusing THz beam possesses a longer focal depth, a larger focal spot, and a slower phase evolution, so that a bigger central dark focus, a thicker light ring, and a smoother Gouy phase shift are generated in the formed THz bottle beam. Actually, the characteristics are similar to the converging process of a THz Gaussian beam^24^. According to the discussion all above, it can be concluded that an axicon with a smaller base angle and a lens with a shorter focal length should be picked as the wave font modulators for forming a more compact optical barrier.

## Discussion

Herein, we still want to simply discuss similarities and differences between THz special beams carrying orbital angular momentum (OAM) and the THz bottle beam. Currently, various THz beams carrying OAM have been paid more and more attentions due to their important application potentials in THz imaging and communications, such as Bessel, Laguerre-Gaussian, and Airy beams with high-order topological charges. Analogous to the THz bottle beam, these THz beams also have a hollow-core intensity^25^. However, the discrepancy between these THz beams and the THz bottle beam is that the central intensity nulls of these THz beams are originated from their phase singularities, so their central zero-amplitude zones are in two dimensions. Meanwhile, the dark focus of the THz bottle beam is due to the interference effect of converging or diverging THz beams so that the THz optical capsule is formed in three dimensions. Therefore, researchers prefer to classify these THz beams carrying OAM as the THz hollow beam. Actually, the optical bottle beam carrying OAM has been also investigated in 2015. Interestingly, the radius of the central annular amplitude is fixed with varying the topological charge for an optical bottle beam carrying OAM, so this kind of optical beam is called as “perfect vortex beam”^21^. To be honest, diffraction characteristics of these special THz beams have been underutilized, which leaves much room for the development of the future THz technology.

In conclusion, the THz bottle beam is generated by utilizing a Teflon axicon and a silicon lens. The complex field of the THz bottle beam are coherently characterized by applying the THz imaging system with a focal-plane array and the evolution process of the THz field is detailedly recorded by implementing the Z-scan measurement. For a linearly polarized THz bottle beam, *E*_*x*_ exhibits the amplitude distribution of a Bessel-like beam and the doughnut-shaped optical barrier on the two terminals and the intermediate section of the optical bottle. Besides, the *E*_*x*_ phase pattern shows the converging as well as diverging processes of the THz beam refracted by the Teflon axicon after passing through the silicon lens and manifests the formation origin of the THz bottle beam. Besides, the *E*_*z*_ component of the THz bottle beam is measured and analyzed by applying the vector measurement function of the THz imaging system. The *E*_*z*_ component with a linear or a circular polarization separately shows a double-lobe characteristics or a vortex pattern. By adopting the vectorial diffraction algorithm, the complex field characteristics of the *E*_*x*_ and *E*_*z*_ components are exactly simulated. Finally, performance tuning of the THz bottle beam is achieved by adjusting the parameters of the Teflon axicon and the silicon lens. The switch of the optical barrier can be easily controlled by varying distance the between the Teflon axicon and the silicon lens. With decreasing the base angle of the Teflon axicon or the focal length of the silicon lens, the THz bottle beam shows a stronger optical barrier and a smaller central dark focus. In a nutshell, this work describes the vector characteristics of the THz bottle beam in detail and achieves the modulations to the features of the THz beam. We consider that the work is helpful for the application and development of the THz technology in particle manipulation and microscopy. In addition, these experimental laws and theoretical discussions can be readily transferred to the infrared, visible, and other frequency ranges.

## Methods

To observe the characteristics of a THz bottle beam, a THz imaging system with a focal-plane array is utilized to acquire the complex field of the THz beam, including amplitude and phase information. Figure 1a presents the schematics of the experimental setup. The light source is a Spectra-Physics femtosecond laser amplifier with a central wavelength of 800 nm, a pulse duration of 50 fs, a repetition ratio of 1 kHz, and an average power of 700 mW. The incoming laser is divided into the exciting and detecting beams for the generation and detection of the THz radiation. The average powers of the exciting and detecting beams are 690 mW and 10 mW, respectively. Firstly, a concave lens (L1) with a focal length of 50 mm is used to expand the exciting beam and a <110> ZnTe crystal with a thickness of 3 mm is chosen as the THz source. After the exciting beam passing through the ZnTe crystal, a THz beam with an x-linear polarization is generated by the optical rectification^26^. Then, the THz beam is collimated by a parabolic mirror (PM) with a focal length of 100 mm for forming a THz quasi-plane wave. The collimated THz beam possesses a diameter of 14 mm. A Teflon axicon and a silicon lens are used as the wave front modulators for generating a THz bottle beam, as shown in Fig. 1b. The incident THz beam successively passes through them to form the peculiar THz field. The out-going THz field illuminates a sensor crystal for detecting the complex THz field. In the path of the detecting beam, a polarizer (P) is used to ensure the probe polarization. The detecting beam is reflected onto the sensor crystal by a non-polarization beam splitter with a 50/50 ratio. In the sensor crystal, the probe polarization is modulated by the THz field to carry the two-dimensional THz information due to the Pockels effect^27^. Then, an imaging module is adopted to receive the reflected detecting beam, which is composed of a lens (L2), a quarter wave plate (QWP), a Wollaston prism (PBS), a lens (L3), and a CCD camera. A mechanical chopper is used to modulate the output frequency of the exciting beam. A balanced detection method^28^ and a dynamics subtraction technique^29^ are utilized to remove the background intensity of the detecting beam. A series of THz temporal images are acquired by adjusting the relative delay between the THz and detecting beams and the THz images in the frequency domain are extracted by operating the Fourier transformation.

To reconstruct the evolution process of a THz bottle beam, the Teflon axicon and the silicon lens are mounted on a motorized translation stage to fulfill a Z-scan measurement. The focal point of the silicon lens is viewed as the base point. The diffraction process of the THz beam is recorded from z = −4.5 mm to z = 4.5 mm and the scanning step is set as 0.5 mm. The advantage of the measurement scheme is that the optical path of the THz beam is fixed, so the linear phase term exp(*jk*z) of the THz wave is negligible. Besides, a quartz TQWP with a central wavelength of 385 μm and a bandwidth of 200 μm is utilized to adjust the THz polarization for observing the discrepancies between the linearly and circularly polarized THz bottle beams.

To comprehensively observe the vector characteristics of a THz bottle beam, the different polarization components need to be separately measured, including the transverse (*E*_*x*_) and longitudinal (*E*_*z*_) components. In the experiment, a ZnTe crystal with a <110> crystalline orientation and a 1 mm thickness is picked up as the sensor crystal to measure *E*_*x*_. The angle between the <001> axis of the crystal and the polarization direction of the detecting beam and is fixed as 0° to maximize the detection efficiency^30^. To acquire the *E*_*z*_ component, a ZnTe crystal with a <100> crystalline orientation and a 1 mm thickness is chosen as the sensor crystal. The angle between the <010> axis of the crystal and the polarization direction of the detecting beam is adjusted as 45° for optimizing the detection efficiency^31^. Herein, it should be noted that both of <110> and <100> ZnTe crystals have the identical detection sensitivities to the THz field^32^.
